# Advancing care: optimizing osteoporosis treatment in the older and oldest old population

**DOI:** 10.1007/s40520-025-02973-1

**Published:** 2025-04-12

**Authors:** Claudia Panait, Patrizia D’Amelio

**Affiliations:** 1 Department of Medicine, Service of Geriatric Medicine & Geriatric Rehabilitation, University of Lausanne Hospital (CHUV), Lausanne, 1011 Switzerland; 2 Riviera-Chablais Hospital (HRC), Geriatrics and Rehabilitation Clinic (CGR), Vevey, 1800 Switzerland

**Keywords:** Osteoporosis, Treatment, Older, Oldest old

## Abstract

Osteoporosis is a critical public health issue, particularly in the “older” (those aged over 75) and “oldest old” population (those aged 85 and above), who are at a heightened risk for fractures and related complications. This article reviews current osteoporosis treatments tailored for these age groups, emphasizing the balance between efficacy and safety, while considering cost/benefit aspects. We discuss pharmacological therapies available nowadays and their respective benefits and risks in the old population, based on the available literature on the subject. Special attention is given to specific features of this age category, like challenges of polypharmacy, physiological changes associated with age, comorbidities and patient adherence. This paper highlights the need for individualised treatment plans that consider the patient’s overall health status, life expectancy and quality of life and the importance of continued innovation and personalized care in managing osteoporosis especially among the “older” population.

## Introduction

Globally, the life expectancy at birth has more than doubled in the past two centuries from 39 years to over 85. Such consistent increments in life expectancy at birth are not only attributable to reductions in premature mortality among children and young adults, but also to improving survival prospects of older adults [1]. However, healthy life expectancy has not increased at the same pace, leading to a significant rise in the incidence of age-associated diseases. In 2019, people were expected to remain healthy until the age of 65.3, while in 2020, this dropped to 62.8 years after the COVID-19 pandemic [2].

Age is the leading predictive factor for the majority of the chronic diseases that account for most of the morbidity, hospitalizations, health costs, and mortality worldwide [3]. Amongst age related diseases associated with increased mortality, disability, loss of independency and increased cost, osteoporosis related fractures play an important role [4, 5]. Osteoporosis affects over 35% of older women and 12% of older men worldwide [6]. In the Western Countries, about 30% of women and 20% of men above 50 years of age will fracture during their lifetime [7]. The absolute number of fractures and particularly of hip fractures increases with age, both in men and women and above all in persons older than 85 years [8].

Hip fractures are associated with the highest morbidity and mortality [9], most commonly affecting adults in their eighties with a cumulative 30-day mortality rate between 5 and 10%, percentage that raises up to 30% over a year postoperative period [10].

Those who survive have high probability of acquiring co-morbid disease and disability; thus, putting a strain on healthcare systems and reducing the quality of life of the old population [11].

Although less frequent than in women, hip fractures in men seem to be more severe, approximately one-third of men who experience a hip fracture die within one year, compared to about one-fifth of women [12–14].

Most patients with hip fracture complain of pain and resulting functional limitations still six months after the fracture [15]. Moreover, in older adults, hip fractures are associated with a four-time increase in the risk of institutionalization within the following year [8]. The percentage of patients moving into long-term care following a hip fracture increases significantly with age, rising from 4% for those aged 70–79 to 14% for those aged 80–89 and to 35.3% for those aged 90–100 years [7].

Nowadays several effective anti-osteoporotic treatments are available to reduce fracture risk, in particular parenteral treatment as zolendronate, teriparatide, denosumab and abaloparatide are effective in reducing the risk of vertebral fractures up to 70% [16], however when talking about the oldest population, evidence-based data on the effectiveness of these treatments are sparse and several barriers to treatment exist amongst health professionals, family caregivers and patients.

This vulnerable segment stands to substantially gain from effective anti-osteoporosis treatment, nevertheless the under-diagnosis and under-treatment of osteoporosis particularly in the older adults is frequent [17].

## “The oldest old” population and anti-osteoporotic treatment

The ageing process, even though a physiological condition, is characterized by structural and functional changes affecting all organs and systems and results in reduced homeostatic capacity. Changes in body composition, hepatic and renal function are responsible for both an increase in the volume of distribution of lipid soluble drugs, and a reduced clearance of lipid and water-soluble drugs. All these changes result in a prolonged plasma elimination half-life. Additionally, significant pharmacodynamic changes occur, generally leading to increased drug sensitivity. The reduced functional reserve itself also leads to an increase in sensitivity by impairing homeostatic compensatory mechanisms [18].

Besides these physiopathological changes, older adults are frequently affected by comorbidities and treated with multiple medications.

Also, social factors, as the presence of a caregiver, may play important role in influencing both access to osteoporosis treatment and adherence to prescribed therapies, particularly among the “oldest-old” population. Caregivers, whether family members or professional aides, assist in overcoming barriers such as mobility limitations, cognitive impairment, and transportation challenges, which often hinder older adults from seeking or following through with medical treatments. Research has shown that caregivers can improve the consistency of treatment adherence by ensuring timely medication administration, attending follow-up appointments, and providing reminders. Conversely, the absence of a caregiver may exacerbate treatment gaps, as frail individuals may struggle to manage complex regimens on their own. However, while caregivers provide essential support, they may also introduce negative aspects, such as overprotection, particularly when caring for patients recovering from fragility fractures. Caregivers sometimes limit the mobilization of individuals who have experienced a hip fracture, which can impede recovery and hinder the patient’s rehabilitation process. Nonetheless, the burden placed on caregivers, especially after a fragility fracture, is substantial, necessitating proper education and resources to mitigate caregiver strain. It is critical for healthcare providers to consider both the benefits and challenges associated with caregiving when assessing the needs of the oldest-old, as interventions to improve osteoporosis care and adherence should not only target the patient but also address the caregiver’s role and well-being. Caregivers should be integrated into bone health education and discharge planning, as they are essential to the successful management of osteoporosis (for a comprehensive review on the role of caregivers in the treatment of osteoporotic patients see the integrative review on Family/caregiver influence on osteoporosis management for older people by Zubick et al. [19].

Therefore, older adults should be considered a distinct population when deciding on treatment strategy.

The threshold settled for being classified as “older” adults has been shifted from 65 years to 75 years recently. Within the cohort of older adults sits a sub-group of ‘the oldest old’ [20]. Several definitions for this group have been proposed: the American Geriatric Society and the World Health Organization define the “oldest old” as individuals aged over 80 years, while the British Geriatrics Society uses 85 years as a threshold. In recent publications, the cut off has been fixed at 85 or 90 years and over [21]. All these groups, especially the oldest old age category, are under-represented in clinical trials [22], for this reason, here we will refer to evidence generated in patients of 75 years or above unless differently specified.

In this population, as mentioned, osteoporosis and osteoporotic fractures are common and place a significant burden on the patients, their families, and the healthcare system. This disease worsens both the remaining quantity and quality of life, while also increasing healthcare costs. However, although several effective treatments for osteoporosis exist, only a small proportion of older individuals are treated, even after major fracture [17].

A population-based study of older adults reported that only 21.8% out of 2,983 eligible older Swedish women received appropriate anti-osteoporosis treatment [23]. Similarly, in the Newcastle 85 + cohort, only 28.6% of 259 individuals identified as needing treatment were on anti-osteoporosis medication [24]. A Greek study showed that patients aged 66–75 years were four times more likely to receive treatment compared to patients older than 86 years [25]. These data are particularly striking when referring to the frail older patients. Among nursing home residents with a history of hip fracture, only 25% received calcium and vitamin D supplements [26]. Regarding active anti-osteoporotic treatment, Rojas-Fernandez and colleagues found that 75% of nursing home residents with osteoporosis were not treated [27].

Paradoxically, the therapeutic care gap may be particularly wide in older, frail patients, for whom the importance and impact of treatment are significant, especially among those living in nursing homes [28]. In older patients who had already experienced a fragility fracture, the clinical need for treatment is even higher, knowing that a history of prior fracture at any site significantly increases the risk for future fractures [29].

Several reasons are advocated for undertreating older patient at high risk of osteoporotic fractures as the lack of evidence base data, presence of polypharmacy and fear for increased adverse events, increased health costs and the risk of therapeutic futility in this population.

## How should we treat osteoporosis in the older population?

It has recently been suggested that the treatment of chronic non-communicable diseases will have a tremendous economic impact by reducing mortality and increasing the quality of life of older individuals [30].

More specifically, it has been proposed that secondary fracture prevention interventions, which increase the prescription of anti-osteoporotic treatments compared to usual care, are highly likely to be cost saving. These interventions could improve future health outcomes and reduce healthcare spending compared to usual care [31].

Despite these general recommendations, questions arise on what kind of intervention must be recommended in the older population. In the following paragraphs we will describe different pharmacological interventions and their efficacy and appropriateness in this category of patients.

### Calcium and vitamin D

The effectiveness of calcium and vitamin D supplementation in preventing osteoporotic fractures remains unclear. Several meta-analyses have reported a reduction in hip and non-vertebral fractures, and possibly also in vertebral fractures in patients taking combined calcium and vitamin D supplements [32–34] while others showed no association between the use supplements that included calcium, vitamin D or both with a lower risk of fractures [35, 36], whereas it is clear that calcium supplementation alone is not effective in preventing fractures [37].

In clinical trials, vitamin D supplements do not improve bone density, except in analyses of subgroups with baseline levels of 25-hydroxivitamin D lower than 30 nmol/L [38, 39], however, in older institutionalized subject at high risk of fracture, adequate vitamin D supplementation may reduce the risk of falling, thus contributing to the falls related-fractures [8]. In the same population, the combination of calcium and vitamin D is beneficial in modestly reducing the risk of non-vertebral fractures and hip fractures [40]. On the other hand, data concerning the fall risk reduction for community dwelling older adults are less convincing [41].

Although several thresholds have been proposed to define optimal levels of 25-hydroxivitamin D [42], it is generally agreed that the optimal plasma level of vitamin D for older subjects should be 75 nmol/L or above [8], whereas serum levels above 100–125 nmol/L must be avoided as this could possibly increase the risk of falls [43].

As concerns calcium intake, the recommended intake for the older adults according to the National Institute of Health is 1200 mg per day, however, generally the calcium intake and the intestinal absorption is decreased in this population [44]. Low calcium intake and vitamin D deficiency lead to secondary hyperparathyroidism and bone loss [45].

In patients treated with antiresorptive drugs, adequate levels of serum vitamin D and calcium will help avoiding hypocalcemia.

In clinical practice, it is recommended to prioritize dietary sources of calcium, as they have been shown to be more effective in promoting bone health compared to calcium supplements. This is likely due to the enhanced absorption of calcium when consumed with meals, as well as the fact that individuals tend to consume smaller amounts more frequently, which aligns better with the body’s natural absorption processes [45]. Supplements should be targeted to those who do not get sufficient calcium from their diet and who are at risk of osteoporosis and/or fragility fracture, such as older adults who are housebound or living in residential or nursing care [46].

Besides calcium and vitamin D supplementation, anti-osteoporotic drugs can be classified into two main categories: anti-resorptive agents, which primarily inhibit bone resorption, and anabolic agents, which mainly stimulate bone formation. The recently approved drug romosozumab is the unique drug with a dual mechanism of action.

Except for raloxifene, which is preferably used in women under 70 years due to its marginal benefits in preventing non-vertebral fractures [47, 48], data show the densitometric and anti-fracture efficacy of anti-osteoporotic treatments in patients over 70, with a precocious efficacy and a favourable benefit/risk ratio in this population [8].

Reduced treatment adherence is a significant challenge, particularly in this population, where polypharmacy, cognitive decline, functional impairments, and adverse drug effects can all negatively impact adherence to osteoporosis treatments. Studies have shown that adherence to oral bisphosphonates decreases significantly over time, with up to 50% of patients discontinuing treatment within the first year [49]. To address this challenge, parenteral options, which have demonstrated higher adherence rates, can be considered as a valid alternative to oral treatments [50].

### Anti-resorptive agents

Bisphosphonates are the most ancient anti-resorptive drugs available for the treatment of osteoporosis, they act by inhibiting bone resorption, are effective in reducing the risk of fractures and are associated with minor side effects. The most frequent side effects are gastrointestinal intollerance and flu-like syndrome [48, 51].

Denosumab is a monoclonal antibody that inhibits bone resorption by blocking RANKL, it has been proved as a safe and effective by the FREEDOM trial, this registration trial enrolled postmenopausal women with a mean age of 72.3 years [52].

In older patients antiresorptives are effective in reducing vertebral and non-vertebral fracture risk, a recent metanalyses showed that the combined effects of antiresorptive agents were significantly more effective than placebo in reducing the risk of vertebral fractures (RR = 0.43; 95% CI = 0.35–0.53; *p* < 0.001) and non-vertebral fractures (RR = 0.84; 95% CI = 0.74–0.96; *p* = 0.009). For hip fracture treatment, a pooled effect of bisphosphonates has been observed (RR = 0.75; 95% CI = 0.58–0.97; *p* = 0.028) in patients older than 75 years [53].

In particular, Alendronate is effective in reducing new vertebral fractures (RR = 0.62) in osteoporotic women aged ≥ 75, as demonstrated by a post hoc analysis of the Fracture Intervention Trial, by Ensrud and colleagues; however, its effect was not significant in reducing clinical fractures [54].

The HIP study demonstrated that risedronate lowers the risk of hip fractures (RR = 0.6) and nonvertebral fractures in women aged 70–79, but was not effective in reducing hip fractures in those older than 80 years [55]. Boonen et al.’s pooled data indicated a benefit of risedronate in reducing vertebral fractures (hazard ratio = 0.56), but not non-vertebral fractures in women over 80 [56]. Eastell et al. observed significant reductions in vertebral fractures with zoledronate in those older than 75 [57]. Similarly, Boonen et al. found that annual zoledronate treatment significantly reduced clinical, vertebral, and nonvertebral fractures in women over 75 years [58].

Ibandronate has been shown to be effective in reducing the risk of vertebral fractures at all ages [59], however, as its efficacy has not been demonstrated for femoral fracture, it should not be considered as a first line treatment in the oldest patients.

Denosumab, in women older than 75 years, was effective in reducing vertebral (RR = 0.36) and hip fractures, though the reduction of non-vertebral fracture risk was not significant. The safety profile was comparable to placebo [60, 61]. A post hoc analysis of the FREEDOM trial carried out to evaluate the effect of denosumab in high-risk populations, among which persons aged ≥ 75 years, showed that denosumab significantly reduced the risk of hip fractures by 62% [60]. The risk reduction for this group age was comparable with the risk reduction in the overall study population by FREEDOM trial [52]. The FREEDOM study showed a reduction of new vertebral fractures by 68% [52]. The analysis of the FREEDOM trial by McClung et al. in 2012 confirmed that denosumab reduces the risk of vertebral fracture to the same extent in subjects aged ≥ 75 years as in subjects aged < 75 years [61].

Before starting a treatment, possible adverse events and contraindications may be considered especially in older frail patients that are comorbid and treated with polypharmacy; renal insufficiency is quite common in older patients and therefore may be considered when choosing an antiresorptive treatment, indeed bisphosphonates are not recommended in patients with severe renal impairment (creatinine clearance < 30–35 mL/min) [17], whereas denosumab is not contraindicated in patients with impaired renal function.

Oral bisphosphonates may cause gastrointestinal symptoms in 20–30% of the patients and zoledronate may induce a flu-like syndrome in about a third of the patients receiving the first injection. Symptoms are usually mild and resolve within 2–3 days [62]. Hypocalcaemia can occur especially in patients with a severe vitamin D deficiency [31].

Other adverse events as osteonecrosis of the jaw, atrial fibrillation and atypical femoral fracture are uncommon [63] and must not be determinant in choosing the treatment; however, in older patients a particular attention to oral health is important to avoid increased risk of the jaw osteonecrosis [64].

Amongst bisphosphonates, the use of the parenteral route, particularly zoledronic acid, in this population with multiple comorbidities and complex treatment regimens is of particular interest. Given the challenges associated with adherence to oral bisphosphonates, including gastrointestinal intolerance, reduced intestinal absorption due to age-related physiological changes [65], and the strict administration requirements, the parenteral route offers a clear advantage. By ensuring full adherence with a single annual infusion, it bypasses the potential issues of diminished drug absorption in the gut, which is especially relevant in the “old” population. Additionally, parenteral administration avoids potential drug interactions at the gastrointestinal level, which is particularly important in polymedicated patients, ensuring better therapeutic outcomes and reducing the risk of fractures.

Beyond these advantages, parenteral drugs also offer a crucial benefit in the context of imminent or very high fracture risk [66], which is particularly relevant in the “oldest old”. The rapid and consistent inhibition of bone resorption achieved through intravenous administration allows for a faster reduction in fracture risk compared to oral treatments, which require prolonged adherence for full efficacy. This is particularly important in frail individuals at immediate risk, where a delay in fracture prevention could lead to severe disability or mortality. Therefore, given both the enhanced adherence and the ability to promptly reduce fracture risk, parenteral bisphosphonates should be considered a preferred option in this highly vulnerable population.

Concerning the use of denosumab, it is associated with severe hypocalcemia in patients with advanced chronic kidney disease, hence a close monitoring and replacement of calcium and vitamin D are required to avoid the development of hypocalcemia in these patients [51]. To mitigate this risk, some authors have proposed the use of calcitriol to enhance intestinal calcium absorption and support calcium homeostasis [67, 68]. While this approach may help reduce hypocalcemia incidence in patients with chronic kidney disease stages 4–5, it requires careful monitoring due to the potential for hypercalcemia and vascular calcifications, especially in those with compromised renal function [69]. Therefore, individualized risk-benefit assessment is crucial when considering vitamin D analog supplementation in this population.

Denosumab is well tolerated with no clincally significant post-dose effects, as for bisphosphonates a mild increase in the risk of osteonecrosis of the jaw and atypical femoral fractures have been reported [70]. Discontinuation of denosumab treatment may be associated with rapid bone loss that may result a rebound effect with the appearance of multiple vertebral fractures, especially in patients with a prior vertebral fracture [71]. For this reason, during periods of suspended treatment, alternative antiresorptive therapy should be considered to maintain bone density gains and prevent the rebound effect [72].

Given that denosumab is often considered for long-term osteoporosis management, it is important to acknowledge the potential for continued use in the “oldest old” population, including those approaching end-of-life care. While there are no specific studies addressing denosumab use in this age group through the end-of-life period, the possibility of maintaining denosumab treatment in this population should be considered carefully, especially in light of its ability to prevent fractures and maintain bone strength. However, clinicians should weigh the potential benefits against the risks of long-term treatment in patients with multiple comorbidities, taking into account their overall prognosis and quality of life.

Transitioning to another anti-resorptive therapy, particularly zoledronate, has shown partial effectiveness in preventing bone loss after denosumab treatment, though study results are inconsistent. Switching to romosozumab, an anti sclerostin antibody, also appears effective in preventing this issue. On the contrary, transitioning to teriparatide, a parathyroid hormone analogue, is associated with significant bone loss in the radius and hip, rendering teriparatide contraindicated. While it remains uncertain whether these transitions fully prevent rebound fractures, observational studies suggest that switching to zoledronate may reduce this risk [31].

Potential benefit of combination therapy between denosumab and teriparatide has been suggested [73], the concurrent administration of denosumab and teriparatide appears to provide superior BMD gains compared to monotherapy or sequential approaches alone. These findings suggest that an overlapping strategy rather than a direct switch could be beneficial in specific patient populations, particularly those at high fracture risk. However, given the limitations of current evidence, further research is necessary to grant the benefit of combination therapy especially in the old population.

In the “oldest old” population the long lasting activity of bisphosphonate is particularly interesting, it is well known that alendronate effects persist for 1–2 years, and effects of zoledronate last for more than 5 years. Sustained fracture prevention was seen in a zoledronate trial lasting 6 years, and some studies suggest that anti-fracture efficacy persists for 3–5 years after discontinuation [31]. The long-lasting effects of bisphosphonates may allow clinicians to offer a “drug holiday” to frail older individuals, thereby reducing the prescription of additional medications in patients already receiving polypharmacy. This is feasible provided that these patients are considered at low or moderate risk of fracture. However, high-risk patients should continue treatment without interruption [74].

The decision to initiate bisphosphonate treatment in frail older patients should carefully consider the time to benefit relative to the patient’s life expectancy to avoid therapeutic futility. Studies indicate that approximately 12.4 months of treatment with a bisphosphonate are required to prevent one non-vertebral fracture [75].

### Anabolic agents

Teriparatide and abaloparatide (the last has been approved by the European Medicine Agency on December 2022) are anabolic agents that stimulate bone formation and activate bone remodeling, both treatements are effective in reducing the risk of vertebral and non-vertebral fractures [76, 77].

In patients aged 75 and older, teriparatide was shown to reduce the incidence of new vertebral fractures (RR = 0.35, *p* < 0.05) and non-vertebral fractures (RR = 0.75, *p* = 0.661). Older patients experienced no more side effects as compared to the younger ones, suggesting that teriparatide was well tolerated in this age group [78, 79]. Also the once-weekly administration of teriparatide significantly reduced the risk of vertebral fractures in patients aged 75 and older (RR = 0.30; 95% CI = 0.11–0.81, *p* = 0.018) [80]. Even in an head to head trial, the VERO study, teriparatide was more effective than risedronate in reducing new vertebral fractures across all age groups [81].

Abaloparatide effectively reduced the incidence of vertebral and nonvertebral fractures, even in the very old patients cohort being safe and well tolerated [82, 83].

As for side effects, only diarrhea was reported more frequently, whereas cataract, deafness, pruritus, and weight loss were reported less frequently in the older compared to the younger age-group [51].

There were more study discontinuations secondary to adverse events in the abaloparatide group than the teriparatide or placebo groups. Nausea, dizziness, headache, and palpitations were the main problems [31].

For both teriparatide and abaloparatide, efficacy data in the oldest old, especially for non vertebral fractures, is substantially limited by sample size and insufficient power. Considering the cost of the medication, need for daily injection, and other potential adverse events, these drugs may be limited to select older adults at very high risk of fractures, particularly vertebral fractures [51, 84].

### Dual action agent

Romosozumab, a monoclonal antibody, is the newest agent with both osteoanabolic and anti-resorptive mechanism approved for the treatment of osteoporosis [84]. This antibody targets and inhibits sclerostin, a protein that naturally inhibits bone formation; by blocking sclerostin, romosozumab promotes both bone formation and reduces bone resorption, leading to an increase in bone density. This dual mechanism makes it unique among osteoporosis treatments. The STRUCTURE phase 3 trial enrolled women up to age 90 (mean age 71 years) with osteoporotic fracture, having received at least 3 years of oral bisphosphonates, and randomized to either romosozumab or teriparatide for 12 months [85]. There was a significant increase in BMD at lumbar spine (+ 9.8% versus + 5.4% with teriparatide), total hip (+ 3% versus − 0.5%), and femoral neck (+ 3.2% versus − 0.2%), however no age stratified data were provided [84].

In the Active Controlled Fracture Study in Postmenopausal Women with Osteoporosis at High Risk (ARCH), the effectiveness of a treatement regimen starting with romosozumab for 12 months followed by alendronate for 12 months was compared to alendronate alone for 24 months. The results showed that romosozumab treatment significantly reduced the risk of fractures compared to alendronate alone. Although the study did not include an age-specific subgroup analysis, 52% of the participants were aged 75 years or older [86].

The Fracture Study in Postmenopausal Women with Osteoporosis (FRAME), compared romosozumab with placebo and included 30% of women in the ≥ 75 years age group (overall population mean age 70.9 years) [87]. In the romosozumab group there was a significant reduction in the incidence of new vertebral fractures (RR = 0.27, 95% CI 0.16–0.47) at 1 year, but no significant reduction in new non-vertebral fractures (HR = 0.75, 95% CI 0.53–1.05). Although a subgroup analysis, including those aged 75 years and older, indicated similar treatment effects, the specific data for this subgroup were not provided [84].

Romosozumab was also studied in men 55 to 90 years with osteoporosis in the placebo-controlled BRIDGE trial, which assessed the efficacy and safety of romosozumab in treating men with osteoporosis [88]. The mean age of the participants was 72.4 years with approximately 40% of subjects aged 75 years or older. A subgroup analysis of men aged 70 years and older showed significant increase in BMD at the lumbar spine (+ 10.2% difference from placebo), total hip (+ 2.8%), and femoral neck (+ 2.6%).

Despite the efficacy data in the older population, Romosozumab has been associated with an increased risk of serious cardiovascular events, hence it should be prescribed in this population only after a careful evaluation of the cardiovascular risk [84].

The main advantages and disadvantages of different therapeutic options available for the treatment of osteoporosis in the older population are summarized in Table 1.

**Table 1:** Advantages and disadvantages of anti-osteoporotic treatment options in population over 75 years

Intervention	Advantages	Disadvantages
*Bisphosphonates*: Alendronate Risedronate Zolendronic acid	Possible parenteral administration (zoledronate) 1 × 12 months Up to 10 years of treatment duration Sustained fracture prevention after discontinuation permitting “drug holydays” in low-moderate risk patients	Gastrointestinal adverse events Use limited to GFR ≥ 30-35 ml/min Limited adherence especially to oral administration forms
*RANKL inhibitor*: Dénosumab	Well tolerated Subcutaneous administration 1 x/6 months Safe for administration up to 10 years Appropriate in the long-term osteoporosis management	High risk of hypocalcaemia especially if GFR < 35 ml/min High risk of rebound vertebral fractures fallowing treatment discontinuation
*Anabolic agents*: Teriparatide Abaloparatide	Well tolerated Indicated in severe osteoporosis	Subcutaneous administration 1x/day High cost Treatment duration limited to 24 months Transition to another treatment recommended following discontinuation
*Dual action agent:* *Sclerostin inhibitor* Romososumab	Parenteral monthly administration	Treatment duration limited to 12 months Transition to another treatment recommended following discontinuation Contraindicated if recent cardiovascular event (< 12 months)

## Falls and falls prevention

Up to 30% of older adults will experience a fall each year, and about 1% of all falls in this population will result in hip fractures [89]. Thus, fall prevention is a critical aspect of managing osteoporosis in the older population, extends beyond bone health and must incorporate environmental modifications, such as ensuring proper lighting, removing tripping hazards, and using assistive devices like handrails and walkers. Additionally, physical therapy programs focused on improving strength, balance, and gait can significantly reduce fall risk. WHO recommends comprehensive falls prevention strategies, including exercise programs, environmental modifications, and health assessments, to address the multifactorial risk factors of falls [90].

Regular monitoring and individualized interventions tailored to each patient’s specific needs and risk factors are necessary to reduce the risk of falls, minimize the risk of fractures, and ultimately decrease the incidence of disability and mortality in this vulnerable population [91].

## When and how should we treat?

As for the younger adults, the decision to start a treatment relies on the risk assessment and must consider accessory factors as: estimated life expectancy, polypharmacotherapy, comorbidity, effects on the quality of life and cost/benefit ratio.

A few indications for treatment and thresholds for intervention in osteoporosis have been proposed in the literature and recommended in guidelines. These include a bone mineral density (BMD) T-score ≤ -2.5, fracture probability-based scores and the presence of a fragility fracture. A low BMD is associated with an increased risk of fracture. However, a BMD T-score of ≤ -2.5 on its own does not fully explain fracture risk [92].

To fully asses fracture risk, measurement of BMD must be integrated with the consideration of other factors associated to increased risk of fracture as low Body mass index, previous fragility fracture (including morphometric vertebral fracture), parental history of hip fracture, current glucocorticoid treatment (any dose, by mouth for 3 months or more), current smoking, alcohol intake 3 or more units daily, rheumatoid arthritis, secondary causes of osteoporosis (including type I diabetes, long-standing untreated hyper thyroidism, untreated hypogonadism/premature menopause (< 45 years), chronic malnutrition/malabsorption, chronic liver disease, non-dialysis chronic renal failure [47].

The utilization of clinical risk factors with or without BMD measurement has been shown to increase the accuracy of osteoporotic fracture risk assessment. Several fracture risk assessment tools that incorporate different clinical risk factors have been developed. Some tools are based on clinical risk factors alone (QFracture) and others can be used with or without BMD measurement (FRAX, Garvan) [92].

Age has to be considered as the proportion of potentially eligible persons for treatment rises from approximately 30–50% with age, largely driven by the prevalence of prior fracture [93].

The Swiss guidelines updated in 2020, have defined four categories of risk (low, moderate, high and very high/imminent). The treatment recommendation is based on risk category, with choice of specific classes of drugs according to the level of the fracture risk. In defined patients at imminent / very high risk, a more aggressive approach with bone forming agents or anabolic is now recommended as first line therapy, although, depending on the country, some reimbursement issues may still be present [66].

In the older and particularly oldest-old population, the prevalence of specific risk factors for fractures, such as Parkinson’s disease, cognitive impairment, sarcopenia and several others [94], must be analysed separately, as these factors are usually not considered in commonly used fracture risk algorithms. Hence, beyond algorithms that estimate fracture risk, a thorough geriatric evaluation must be conducted to assess the risk of falling, quality of life, functional status, and remaining life expectancy to establish the treatment indication.

## Barriers to treatement implementation and how to overcome them

It has been observed that pharmaceutical prevention of secondary fractures in persons older than 80 years is rare when compared with younger ones and several reasons can be advocated to explain this disparity in treatment. Uncertainty regarding treatment effectiveness, concerns about polypharmacy, frailty, limited life expectancy, perspectives on natural ageing and reduced patient self-advocacy in the older age are factors that may partly explain this discrepancy [95]. Moreover, there is clinicians’ reluctancy to prescribe treatment due to doubts about its effectiveness over a short period [56].

As shown in this article, there is evidence of efficacy in the older population and there are clinically significant benefits in terms of fracture reduction within the first year of treatment [17]. The results of the study conducted by Ström et al. [95] indicate that osteoporosis medication is effective in women older than 80 years, with the magnitude of the observed effect being similar to that estimated in younger women and reported in randomized trials.

A Systematic Review and Meta-Analysis by Wang et al. [53] showed that both antiresorptive and anabolic agents had significant effects on reducing vertebral, nonvertebral, or hip fracture risk, and were well-tolerated by older patients.

The main issues concerning treatment in this population include reduced intestinal absorption (thus lower bioavailability of oral treatments), metabolism (lower metabolic rate), excretion (impaired renal function), tissue sensitivity (skin effects like eczema or allergic skin reactions), concomitant deficiencies (e.g., reduced endocrine responses to GH and PTH), and concomitant treatments (invoking interactions for drug metabolism as well as target organ effects). The large RCTs and meta-analyses have shown however that under relatively stringent conditions, the adverse events tend to be mild to moderate and reversible [17].

In terms of costs a pharmaceutical anti-osteoporosis treatment can change from being cost-effective to being cost saving in the older population with a rational strategy of patient identification [96]. Using a Markov cohort modelling analysis, Lippuner and colleagues estimated the costs associated with the treatment of patients in Switzerland whose fracture risk was estimated using 2 FRAX-based approaches, with the willingness-to-pay threshold for one Quality adjusted Life-Year (QALY) was set at twice the gross domestic product per capita and a first-line treat ment with alendronate (original molecule). The analysis found that treatment was cost-effective in women having a 10-year risk for a major osteoporotic fracture of 13.8% or more, whereas for men the risk estimate should exceed 15%. Using a translational approach, i.e. the equivalence to a prevalent spine or hip fracture, it was found that in individuals having a previous fragility fracture, treatment was cost-effective in women aged over 60 years and in men aged over 55 years, and cost-saving above the age of 75 years [17].

Thus, starting treatment with an anti-osteoporosis drug would be beneficial in terms of efficacy, safety, and costs and it would contribute to an improved quality of life in the older population. A skilled primary care provider, along with effective patient education, could play a key role in conveying the seriousness of the disease, its potential consequences, and the importance of adhering to treatment.

## Conclusion

Osteoporosis is a common metabolic disease and osteoporosis-related fractures are an important cause of morbidity and mortality in older adults. Despite the availability of effective treatments, there is a treatment gap, especially in the older and oldest population.

As this condition increases proportionally with age and life expectancy continues to rise, older individuals, especially those aged over 85 years old become a key target for detection and treatment. This age group often referred to as the “oldest old” experiences osteoporosis at a heightened prevalence due to age-related physiological changes, including reduced bone density, diminished calcium absorption, and hormonal alterations. These factors, combined with the natural aging process and often compounded by comorbidities and reduced mobility, make this population particularly vulnerable to the debilitating effects of osteoporosis.

Addressing osteoporosis in this demographic segment is crucial for maintaining their functional independence, reducing fracture-related morbidity and mortality, and enhancing their overall quality of life.

Evidence consistently supports both the effectiveness of osteoporosis treatments in reducing fracture risk and its safety even in the “old” and the “oldest old” population, with manageable side effects that do not outweigh the substantial benefits.

From an economic perspective, investing in osteoporosis treatment is prudent; it may reduce the long-term costs associated with fracture management and subsequent healthcare needs.

In conclusion, treating osteoporosis in the “older” and “oldest old” population is not only crucial, but also highly beneficial as it improves longevity and well-being while optimizing healthcare resources. The key messages of this paper are summarized in the box 1.

## Box 1. Paper’s key messages

**Table:** 

Key Messages
• Osteoporosis is a major public health concern in individuals aged 75 years and older, with a significant impact on morbidity, mortality, and healthcare costs.
• Despite the availability of effective treatments, osteoporosis remains underdiagnosed and undertreated, particularly in the oldest old, leading to preventable fractures and disability.
• Treatment decisions should be individualized, considering life expectancy, polypharmacy, renal function, fall risk, and the potential for therapeutic benefit.
• Anti-resorptive agents (bisphosphonates, denosumab) and anabolic therapies (teriparatide, abaloparatide, romosozumab) have demonstrated efficacy in reducing fracture risk in older adults, with manageable safety profiles.
• Injectable osteoporosis treatments (e.g., zoledronate, denosumab) offer advantages in adherence and fracture prevention, particularly in frail older adults with complex medication regimens.
• Optimal osteoporosis management in this population requires a combination of pharmacological treatment, fall prevention strategies, caregiver involvement, and healthcare provider education.

## Data Availability

No datasets were generated or analysed during the current study.
